# Dietary Quercetin Attenuates Adipose Tissue Expansion and Inflammation and Alters Adipocyte Morphology in a Tissue-Specific Manner

**DOI:** 10.3390/ijms19030895

**Published:** 2018-03-17

**Authors:** Laura A. Forney, Natalie R. Lenard, Laura K. Stewart, Tara M. Henagan

**Affiliations:** 1 Nutrient Sensing and Adipocyte Signaling Pennington Biomedical Research Center, Baton Rouge, LA 70808, USA; laura.bobart@pbrc.edu; 2 Biology Department, Franciscan Missionaries of Our Lady University, Baton Rouge, LA 70808, USA; natalie.lenard@franu.edu; 3 School of Sport and Exercise Sciece, University of Northern Colorado, Greeley, CO, 80639, USA; laura.stewart@unco.edu; 4 Nutrition Science, Purdue University, West Lafayette, IN 47907, USA

**Keywords:** quercetin, inflammation, browning, mulilocular adipocyte, red onion extract, botanical extract, adipose tissue, adipocyte, adipokine

## Abstract

Chronic inflammation in adipose tissue may contribute to depot-specific adipose tissue expansion, leading to obesity and insulin resistance. Dietary supplementation with quercetin or botanical extracts containing quercetin attenuates high fat diet (HFD)-induced obesity and insulin resistance and decreases inflammation. Here, we determined the effects of quercetin and red onion extract (ROE) containing quercetin on subcutaneous (inguinal, IWAT) vs. visceral (epididymal, EWAT) white adipose tissue morphology and inflammation in mice fed low fat, high fat, high fat plus 50 μg/day quercetin or high fat plus ROE containing 50 μg/day quercetin equivalents for 9 weeks. Quercetin and ROE similarly ameliorated HFD-induced increases in adipocyte size and decreases in adipocyte number in IWAT and EWAT. Furthermore, quercetin and ROE induced alterations in adipocyte morphology in IWAT. Quercetin and ROE similarly decreased HFD-induced IWAT inflammation. However, quercetin and red onion differentially affected HFD-induced EWAT inflammation, with quercetin decreasing and REO increasing inflammatory marker gene expression. Quercetin and REO also differentially regulated circulating adipokine levels. These results show that quercetin or botanical extracts containing quercetin induce white adipose tissue remodeling which may occur through inflammatory-related mechanisms.

## 1. Introduction

High fat diet (HFD)-induced obesity is accompanied by chronic inflammation in adipose tissue, which may contribute to adipose tissue expansion and insulin resistance [1,2,3,4]. During HFD-induced obesity, adipose tissue expansion is marked not only by an increase in white adipose tissue mass but also by morphological changes which affects adipose tissue function or quality [5]. For example, adipocyte hypertrophy alters adipokine secretion, is associated with a pro-inflammatory state and may itself be sufficient in causing insulin resistance [6,7,8,9]. Additionally, adipocyte hypertrophy in visceral but not subcutaneous adipose tissue is associated with metabolic perturbations in lipid metabolism [10] and accumulation of visceral adipose tissue has been shown to confer higher metabolic dysfunction risk in comparison to subcutaneous adipose tissue accumulation [11,12]. 

In contrast to adipose tissue expansion and adipocyte hypertrophy, induction of adipose tissue remodeling that results in proliferation and differentiation of multilocular adipocytes is associated with decreased adipose tissue mass and improved metabolic function and insulin sensitivity [13,14,15,16,17,18]. The presence of multilocular or “beige” adipocytes in white adipose tissue is termed browning due to the fact that these adipocytes are morphologically and functionally similar to those found in brown adipose tissue. Namely, they are associated with increased nonshivering thermogenesis and energy expenditure and improved metabolic outcomes, including attenuation of HFD-induced obesity and white adipose tissue expansion [14,19,20]. Browning of adipose tissue occurs in both visceral and subcutaneous white adipose tissues; however, a larger degree of browning has been noted in subcutaneous white adipose tissue in response to various environmental stimuli, including cold exposure, exercise and dietary manipulations such as methionine restriction [20,21,22,23,24]. Browning is also associated with alterations in inflammatory gene expression [17,25,26,27]. Thus, it has been proposed that expansion or activation of white adipose tissue browning may improve metabolic outcomes, such as amelioration of insulin resistance. 

Quercetin is a polyphenol that ameliorates HFD-induced obesity and insulin resistance by attenuating HFD-induced white adipose tissue expansion and upregulating energy expenditure, mitochondrial biogenesis and function and completeness of fatty acid oxidation [28]. Dietary quercetin supplementation also decreases systemic chronic inflammation [29]. More recently, dietary quercetin supplementation, without or without the addition to resveratrol, has been shown to induce browning in retroperitoneal white adipose tissue in HFD fed mice [24]. Additionally, quercetin, alone or in association with other phytochemicals, is antiadipogenic, which may contribute to its amelioration of HFD-induced obesity through limiting adipose tissue expansion [30,31]. Thus, the present study aimed to determine the effects of quercetin and red onion extract (ROE) in HFD fed mice on subcutaneous vs. visceral adipose tissue morphology and inflammation as well as circulating adipokine levels, which may contribute to quercetin-induced attenuation of HFD-induced obesity and insulin resistance. 

## 2. Results

### 2.1. Adipose Tissue Phenotype

**Figure 1:**
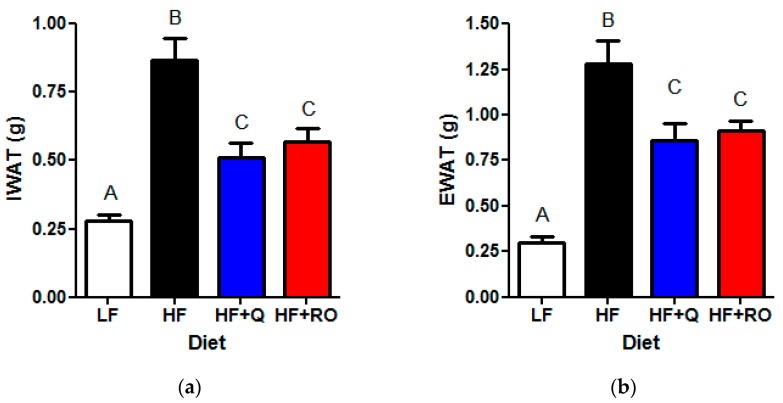
Mice (*N* = 10/group) were fed low fat (LF; white), high fat (HF; black), high fat + quercetin (HF + Q; blue) or high fat + ROE (HF + RO; red) diets for 9 weeks and (**a**) inguinal white adipose tissue (IWAT) and (**b**) epididymal white adipose tissue (EWAT) depots were extracted and weighed. Graphs are mean ± SEM. Different letters denote significant differences between groups at *p* < 0.05.

We have previously published that 9 weeks of dietary supplementation with quercetin (50 μg/day) or ROE containing similar quercetin equivalents significantly ameliorates HFD-induced obesity and insulin resistance [28]. Here, we show that mice consuming HFD (HF) for 9 weeks had significantly larger adipose tissue mass in both the subcutaneous, inguinal, white adipose tissue (IWAT) and visceral, epididymal, white adipose tissue (EWAT) depots compared to low fat fed mice (LF; Figure 1a,b). Dietary supplementation with either quercetin or ROE supplementation similarly significantly attenuated the effect of high fat feeding on IWAT and EWAT size (Figure 1). Despite the significantly smaller adipose tissue depot size, high fat diet fed mice receiving quercetin (HF + Q) or red onion extract ROE (HF + RO) exhibited significantly larger adipose tissue size compared to LF (Figure 1a,b). Thus, quercetin and ROE similarly attenuated HFD-induced adipose tissue expansion in both subcutaneous and visceral adipose tissue depots, and created an intermediate phenotype between that seen in LF and HF mice.

### 2.2. Adipocyte Size and Number

**Figure 2:**
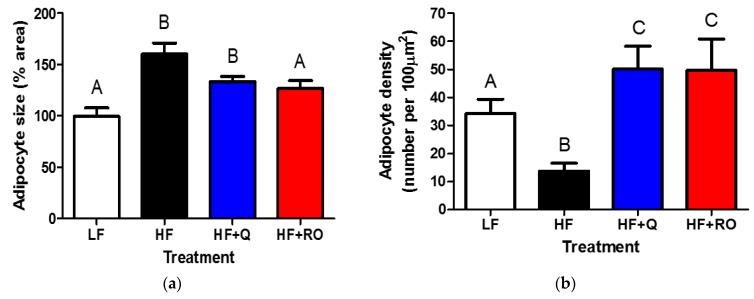

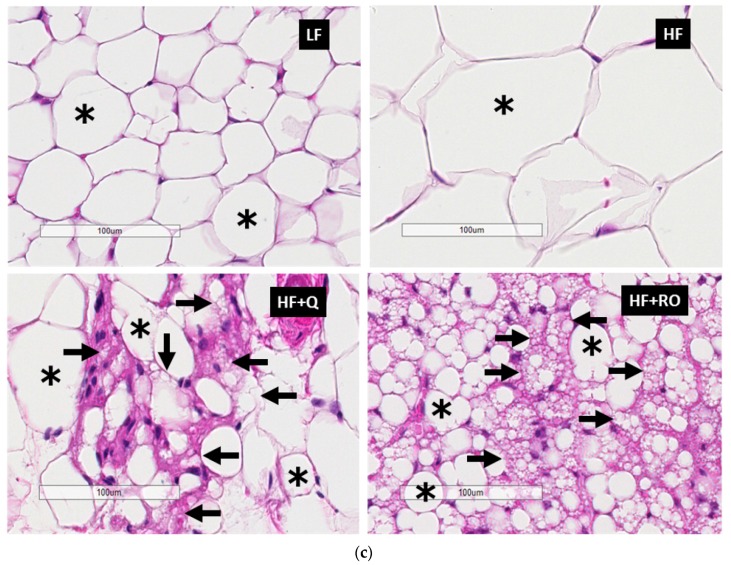
Adipocyte size (**a**) as the percent area, or the number of adipocytes in a given area of tissue, and density (**b**) were determined in inguinal white adipose tissue (IWAT) from mice were fed low fat (LF; white), high fat (HF; black), high fat + quercetin (HF + Q; blue) or high fat + ROE (HF + RO; red) diets and representative images are shown (**c**). Graphs are mean ± SEM. Different letters denote significant differences between groups at *p* < 0.05. Asterisks represent unilocular adipocytes and arrows point to multilocular adipocytes.

Within IWAT, adipocyte size was significantly larger in HF compared to LF mice (Figure 2). Both quercetin and ROE supplementation prevented HFD-induced enlargement in IWAT adipocyte size (Figure 2a,c). IWAT adipocyte density was significantly lower in HF compared to LF (Figure 2b,c); and this lower adipocyte density was prevented in both HF + Q and HF + RO (Figure 2b,c). In fact, both quercetin and ROE supplementation caused significantly greater IWAT adipocyte density in comparison to LF mice (Figure 2b,c). Furthermore, both HF + Q and HF + RO exhibited multilocular adipocytes, which may be indicative of browning of white adipose tissue (Figure 2c). 

**Figure 3:**
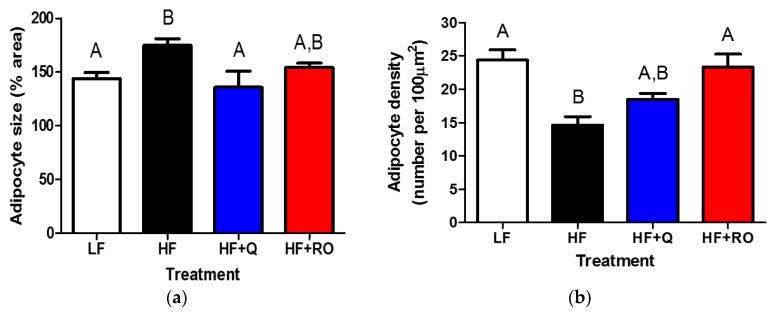

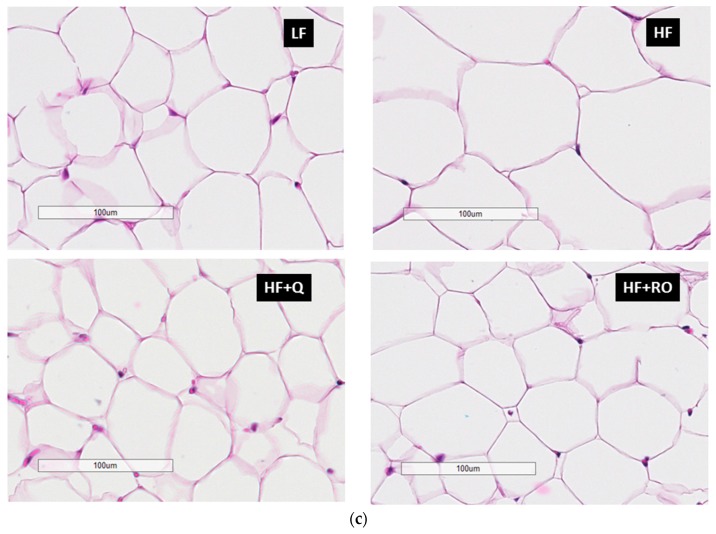
Adipocyte size (**a**) as the percent area, or the number of adipocytes in a given area of tissue, and density (**b**) were determined in epididymal white adipose tissue (EWAT) from mice were fed low fat (LF; white), high fat (HF; black), high fat + quercetin (HF + Q; blue) or high fat + ROE (HF + RO; red) diets and representative images are shown (**c**). Graphs are mean ± SEM. Different letters denote significant differences between groups at *p* < 0.05.

Within EWAT, adipocyte size was significantly larger in HF compared to LF mice (Figure 3a,c). Quercetin but not ROE supplementation prevented HFD-induced enlargement in EWAT adipocyte size (Figure 3a,c). EWAT adipocyte density was significantly lower in HF compared to LF (Figure 3b,c); and this was prevented in HF+RO (Figure 3b,c). HF + Q showed no difference in EWAT adipocyte density in comparison to either LF or HF mice (Figure 3b,c).

### 2.3. Adipose Tissue Inflammation

**Figure 4:**
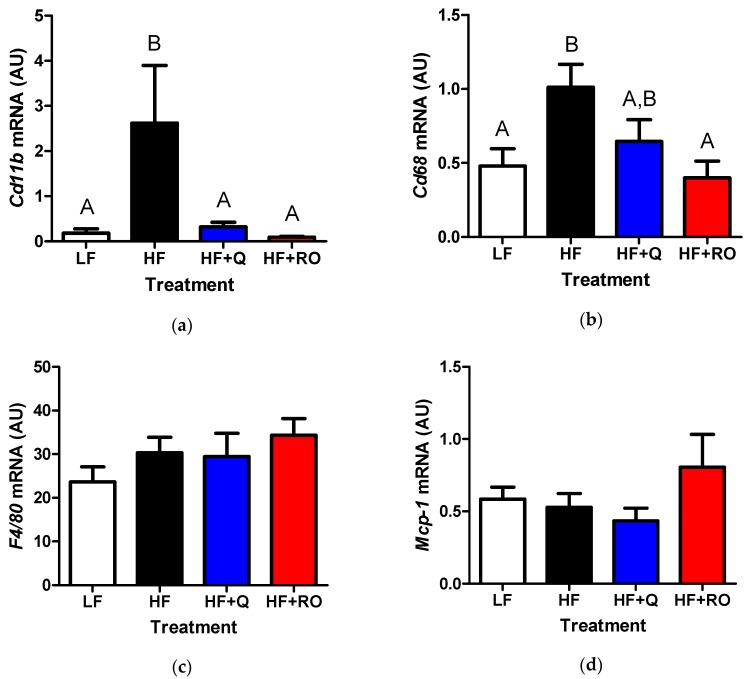
Gene expression for inflammatory markers (**a**) *Cd11b* (**b**) *Cd68* (**c**) *F4*/*80* and (**d**) *Mcp*-*1* were determined in inguinal white adipose tissue (IWAT) from mice were fed low fat (LF; white), high fat (HF; black), high fat + quercetin (HF + Q; blue) or high fat + ROE (HF+RO; red) diets and graphs of respective means ± SEM are shown as arbitrary units (AU). Different letters denote significant differences between groups at *p* < 0.05.

Chronic inflammation has been noted in adipose tissue of obese and insulin resistant states and may contribute to adipose tissue inflammation in response to HFD feeding. Thus, gene expression of several pro-inflammatory markers of chronic inflammation, including *Cd11b*, *Cd68*, *F4*/*80* and *Mcp*-*1*, were measured in IWAT and EWAT. Within IWAT, *Cd11b* was significantly higher in HF compared to LF (Figure 4a). This elevation was completely abolished in HF + Q and HF + RO, creating an expression level similar to that seen in LF (Figure 4a). Similarly, both *Cd68* and *F4*/*80* gene expression were significantly higher in HF IWAT compared to LF IWAT (Figure 4b,c). This elevation was prevented in HF + RO and tended to be ameliorated in HF + Q, which was not different from LF or HF (Figure 4b,c). There was no difference in any groups in IWAT *Mcp*-*1* (Figure 4d).

**Figure 5:**
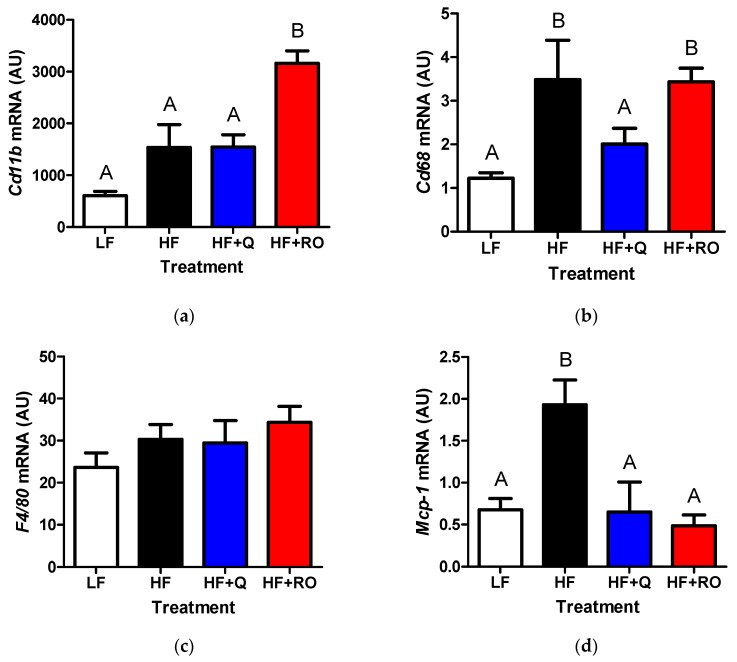
Gene expression for inflammatory markers (**a**) *Cd11b* (**b**) *Cd68* (**c**) *F4*/*80* and (**d**) *Mcp*-*1* were determined in epididymal white adipose tissue (EWAT) from mice were fed low fat (LF; white), high fat (HF; black), high fat + quercetin (HF + Q; blue) or high fat + ROE (HF + RO; red) diets and graphs of respective means ± SEM are shown as arbitrary units (AU). Different letters denote significant differences between groups at *p* < 0.05.

Unlike the inflammatory expression profile observed in IWAT, there was no difference in gene expression of *Cd11b* between LF, HF or HF + Q in EWAT (Figure 5a). Interestingly, *Cd11b* was significantly higher in HF + RO in comparison to all other treatment groups (Figure 5a). However, HFD feeding caused significantly higher expression of *Cd68* in EWAT in comparison to low fat fed mice (Figure 5b). This higher expression was prevented in HF + Q but not in HF + RO, with levels in HF + Q being similar to that observed in LF and levels in HF + RO similar to that in HF (Figure 5b). There was no significant difference in *F4*/*80* between any groups in the EWAT (Figure 5c). Unlike the expression seen in IWAT, EWAT *Mcp*-*1* was significantly higher in HF compared to LF (Figure 5d). Both quercetin and ROE supplementation prevented HFD-induced elevation in EWAT *Mcp*-*1*, restoring *Mcp*-*1* levels similarly to those seen in LF (Figure 5d). 

Collectively, these data indicate that quercetin and ROE may act similarly in subcutaneous IWAT to prevent HFD-induced inflammation. However, quercetin and ROE may act through differential mechanisms to limit HFD-induced white adipose tissue expansion in EWAT.

### 2.4. Systemic Adipokines and Interleukin-6 (IL-6)

**Figure 6:**
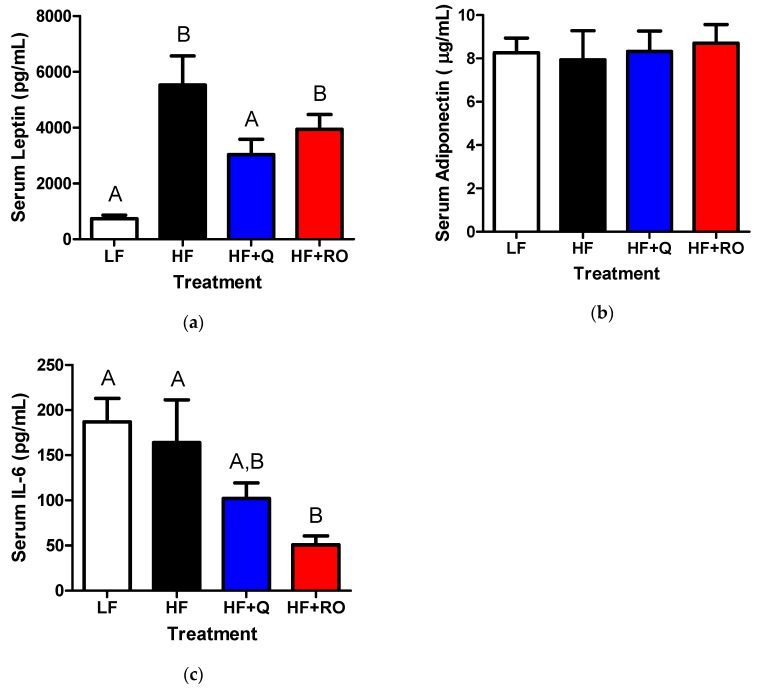
Serum levels of (**a**) leptin (**b**) adiponectin and (**c**) IL-6 were determined in serum from mice were fed low fat (LF; white), high fat (HF; black), high fat + quercetin (HF + Q; blue) or high fat + ROE (HF + RO; red) diets and graphs of respective means ± SEM are shown. Different letters denote significant differences between groups at *p* < 0.05. A is the protein leptin. B is adiponectin and C is IL-6.

Chronic adipose tissue inflammation may be partially regulated by adipokines and chemokines that act to recruit or prevent recruitment of leukocytes. As expected with larger adipose tissue mass, leptin levels were significantly higher in HF compared to LF (Figure 6). This HFD-induced elevation was ameliorated in HF + Q and HF + RO (Figure 6a). There was no difference in adiponectin between any groups (Figure 6b). Interestingly, there was no difference in serum IL-6 levels between LF and HF (Figure 6c). Yet, IL-6 was significantly lower in HF + RO but not in HF + Q compared to both LF and HF (Figure 6c). 

## 3. Discussion

We have previously published that 8–9 weeks of dietary supplementation with 50 µg/day quercetin or ROE containing similar quercetin equivalents significantly and similarly ameliorates HFD-induced obesity and insulin resistance by attenuating percent body fat gain and maintaining lean mass without changing caloric intake [28]. Here, we show that these observed decreases in HFD-induced adipose tissue expansion are partially a result of smaller IWAT and EWAT masses. Furthermore, both quercetin and ROE supplementation lead to lower visceral (epididymal) and subcutaneous (inguinal) white adipose tissue masses, to similar extents. Increases in visceral but not subcutaneous adipose tissue mass are associated with negative metabolic outcomes, including insulin resistance and type 2 diabetes [2]; and, it has been shown by several investigators that quercetin supplementation ameliorates insulin resistance [32,33]. It is also possible that adipose tissue physiology, in addition to tissue mass, may be a large determinant of metabolic dysfunction [34]. Consistent with this hypothesis, Arias et al. 2014 showed that quercetin supplementation improved insulin sensitivity without affecting adipose tissue mass [32]. Although we do see an effect on adipose tissue mass in the present study and previously [28,33], we also note in the present study that both quercetin and ROE altered the morphology and physiology or the “quality” of the white adipose tissue. 

As expected in mice fed the high fact diet, adipocyte size was larger while density was lower compared to low fat fed mice in IWAT and EWAT. Both quercetin and ROE supplementation were able to prevent HFD-induced alterations in the adipocyte morphology and density in IWAT. In both HF + Q and HF + RO we noted the presence of multilocular adipocytes, which may be indicative of white adipose tissue browning or remodeling of adipocytes to a brown adipose tissue-like adipocyte [35], in the IWAT. Similar browning has been noted in subcutaneous IWAT in response to cold exposure [16,36] and dietary methionine restriction [15,20]. Although adipocyte size was also smaller in HF + Q and tended to be smaller in HF + RO EWAT compared to HF, so that adipocyte size was similar to the size seen in LF, and EWAT adipocyte density was greater in HF + RO and tended to be greater in HF + Q compared to HF, again similar to the density observed in LF EWAT, multilocular adipocytes indicative of browning were not observed in the visceral EWAT in response to quercetin or ROE supplementation. Thus, remodeling of the white adipose tissue leading to the presence of multilocular adipocytes, which may be indicative of browning, occurred in a tissue-specific manner. This is similar to what others have reported following other environmental exposures that induce white adipose tissue browning [14,35,37]. Interestingly, it has recently been published that quercetin supplementation or a combination of resveratrol + quercetin supplementation leads to browning in retroperitoneal, but not epididymal or inguinal, white adipose tissue of rats fed a HFD [24]. Of note, the dose of quercetin used in this study was at 30 mg/kg/day and the duration of supplementation was 6 weeks [24]. 

Alterations in the morphology and physiology or “quality” of white adipose tissue occurs in association with alterations in gene expression of inflammatory markers, including *Mcp*-*1*, *F4*/*80*, *Cd11b* and *Cd68* [37]. In IWAT, 5 days of cold exposure leading to white adipose tissue browning does not affect *Mcp*-*1* or *Cd68* expression but significantly decreases expression of *F4*/*80* and *Cd11b* [37]. However, in EWAT, cold exposure had no effect on expression of *Mcp*-*1*, *F4*/*80* or *Cd11b* but increased *Cd68* [37]. Interestingly, we found here that HF + RO but not HF + Q lead to higher expression levels of EWAT inflammatory markers, *Cd11b* and *Cd68* but did not alter *F4*/*80* or *Mcp*-*1*. Higher *Cd68* expression in HF + RO mimicked the higher levels in this inflammatory marker seen in high fat fed animals in comparison to low fat fed animals. However, the higher expression seen in *Cd11b* that occurred in HF + RO was not observed in HF compared to LF mice. Interestingly, *Cd11b* deficiency has been shown to increase EWAT mass and inflammation in high fat fed mice [38]. Thus, it is possible that upregulation of *Cd11b* may have the opposite effect, and that the elevated *Cd11b* observed in HF + RO EWAT may partially contribute to lower EWAT mass and provide a differential mechanism of action between quercetin and red onion extract in ameliorating HFD-induced obesity. Because HFD-induced obesity is associated with adipose tissue inflammation, it is not surprising that we noted elevated levels of *Cd68* and *Mcp*-*1* in high fat fed animals in the present study. Given the insulin sensitizing effects of quercetin and ROE, we did expect a decrease in these inflammatory factors in response to dietary supplementation. However, given the effects of other stimuli which induce adipose tissue remodeling and improve insulin sensitivity, the observed higher levels of EWAT inflammatory markers in HF + RO is consistent with previous reports that cold exposure increases EWAT inflammation [37]. More surprising is the lack of induction of inflammation in HF + Q EWAT despite alterations in the tissue quality. Differences in HF + Q and HF RO EWAT inflammatory gene expression may be due to the slight differences in adipocyte size and density between these treatment groups. While adipocyte size was lower in both groups compared to HF, it was only significantly lower in HF + Q; and while adipocyte density was increased in both groups, it was only significantly higher in HF + RO. Thus, HF + RO had a larger density of hypertrophic adipocytes in comparison to HF and HF + Q, although these hypertrophic adipocytes were not as large as those observed in HF. Also consistent with Luo et al. 2016 [37], we found here that IWAT inflammatory markers, including *Cd11b*, *Cd68* and *F4*/*80* but not *Mcp*-*1*, tended to be lower or were significantly lower in both HF + Q and HF + RO groups, which appeared to exhibit a browning phenotype in this adipose tissue depot. These changes mimicked similar changes in adipocyte size and density between HF + Q and HF + RO.

It has also been reported that cold exposure significantly decreases circulating leptin levels without altering adiponectin levels [37]. Consistent with this stimulus, we observed significantly lower serum leptin levels in HF + Q and nonsignificantly lower levels in HF + RO compared to HF. There was no change in adiponectin between any groups. Leptin is known to be secreted in proportion to adipose tissue size and may induce insulin resistance [39]. Thus, it is not surprising in the present study that we observed higher leptin levels in high fat fed mice compared to low fat fed mice and lower levels in quercetin supplemented mice compared to high fat fed mice. However, we observed no difference between leptin levels in HF and HF + RO mice, despite the significantly smaller adipose tissue mass in HF + RO mice. Thus, it is possible that the adipose tissue mass alone is not the only driving factor in determining serum leptin levels, but adipose tissue quality may also have an effect. Indeed, adiponectin is also known to be secreted indirectly proportional to adipose tissue mass, yet we observed no difference in serum adiponectin levels between any groups in the present study. 

Interestingly, while lower leptin levels in HF + RO did not reach statistical significance, we did observe a significant lower serum levels of the pro-inflammatory cytokine IL-6 in HF + RO. Lower levels were not observed in HF + Q. IL-6 has been shown to chronically upregulated in the obese and insulin resistant state, and has been proposed to contribute to tissue-specific insulin resistance [34]. It is secreted by adipocytes as well as other cell types, such as stromovascular cells and leukocytes in adipose tissue [40,41,42]. Higher levels of IL-6 are produced by subcutaneous rather than visceral adipose tissue, although obesity results in increased visceral IL-6 levels [43]. Interestingly, red onion supplementation decreased IWAT inflammatory marker gene expression to a larger extent than quercetin supplementation. This is consistent with the observed lower levels in the pro-inflammatory adipokine IL-6 in HF + RO but not in HF + Q. Although HFD feeding lead to higher adipose tissue mass and adipocyte hypertrophy and hypoplasia in IWAT and EWAT, it did not affect serum IL-6 levels. Thus, it is possible that upregulation of IL-6 may not be causative in HFD-induced insulin resistance but persistent decreases in IL-6 may be sufficient to improve insulin sensitivity in mice fed HFD.

In conclusion, these results show for the first time that a low dose of quercetin or botanical extracts containing quercetin induces white adipose tissue remodeling in subcutaneous adipose tissue while also limiting HFD-induced hypertrophy and hypoplasia in visceral adipose tissue. Quercetin and ROE-induced adipose tissue remodeling may occur through differential mechanisms related to induction or prevention of a pro-inflammatory state in a tissue-specific manner. 

## 4. Materials and Methods 

### 4.1. Animals and Diets

The protocols for animal and diets have been previously published [28]. Briefly, C57BL/6J mice (Jackson Laboratories, Bar Harbor, MN, USA) were weaned onto low fat diet (LF; Research Diets 12450B, 10% kcal fat) at 5 weeks of age for 1 week. Mice were then randomized into the following dietary treatment groups (*N* = 10/group): LF (Research Diets 12450B, 10% kcal fat); high fat (HF; Research Diets D12451, 45% kcal fat); HF + Q (Research Diets D08072305, 45% kcal fat) with 17 mg/kg quercetin aglycone (Enzo Life Technologies ALX-385-001-G005; Farmingdale, NY, USA); or HF + RO (Research Diets D08072306, 45% kcal fat) with ROE prepared as previously described to yield 17 mg/kg quercetin equivalents [28]. 48 h food consumption and body weight and composition via nuclear magnetic resonance (Bruker Minispec, Billerica, MA, USA) were assessed weekly. After 9 weeks of feeding, mice were euthanized and trunk blood, IWAT and EWAT were collected. IWAT and EWAT were weighed and immediately snap frozen in liquid nitrogen or fixed in 5% paraformaldehyde for further analyses. Ethylenediaminetetraacetic acid (EDTA)-treated blood samples were centrifuged at 4 °C for 20 min at 2000× *g* and serum was collected and frozen for later analyses. All experiments were reviewed and approved by the Pennington Biomedical Research Center Institutional Animal Care and Use Committee (IACUC protocol 799, approved 12 December 2012). 

### 4.2. Immunohistochemistry

All immunohistochemistry was performed by the Cell Biology, Cell Imaging and Cell Culture Core at the Pennington Biomedical Research Center, Baton Rouge, LA, USA. Briefly, fixed tissues were paraffin embedded, sectioned at 5 μm and hemotoxylin and eosin (H&E) stained using a standard protocol [44]. Slides were then scanned on a NanoZoomer digital slide scanner (Hamamatsu, Bridgewater, NJ, USA). All downstream analysis of cell size and number were performed using ImageJ software (version 1.51n, US National Institutes of Health, Bethesda, MD, USA). For analysis of cell size, 2–3 areas in 2 sections from each animal (*N* = 5–6 animals/treatment group) were averaged. For analysis of cell density, or the number of cells per cross sectional area, averages were used form 2 areas in 2 sections from each animal (*N* = 5 animals/treatment group).

### 4.3. RNA Isolationa and Real Time qPCR

Total RNA was isolated from frozen IWAT and EWAT samples, as previously described. Briefly, frozen tissue was homogenized in Trizol reagent and phase separation was carried out using chlorofom per the manufacturer’s protocol (Sigma Aldrich, St. Louis, MO, USA). 70% ethnaol was added to the aqueous layer, which was then further purified using a RNeasy mini kit (Qiagen, Valencia, CA, USA) per the manufacturer’s protocol. RNA quantity and quality were analyzed by spectrophotometry (NanoDrop, ND-1000, Thermo Scientific, Wilmington, DE, USA), and a cDNA library was created using M-MLV reverse transcriptase (Promega, Madison, WI, USA) per the manufacturer’s protocol.

qRT-PCR was performed using commercially available Applied Biosystems Taqman primers and probes (*Cd11b*, Mm01271255_m1; *Cd68*, Mm04411920_m1; *F4*/*80*, Mm00802527_m1; *Mcp*-*1*, Mm00441242_m1; Applied Biosystems, Foster City, CA, USA) and samples were run in duplicate on the ABI 7900HT platform (Applied Biosystems, Foster City, CA, USA) using ABI Taqman Universal MasterMix (Applied Biosystems, Foster City, CA, USA). Gene expression was analyzed using a standard curve and normalization to cyclophilin as the endogenous control. Data are expressed as arbitrary units (AU).

### 4.4. Serum Assays

Commercially available ELISA assay kits (ALPCO Diagnostics, Salem, MA, USA) were used to measure circulating concentrations of inflammatory markers in duplicate from serum samples. Leptin, adiponectin and IL-6 levels were determined using the BioTek platform (Winooski, VT, USA) platform and a standard curve per the manufacturer’s protocols. Each sample was run in duplicate (*N* = 10/treatment group).

### 4.5. Statistical Analyses

All data were analyzed using *N* = 10/group unless otherwise noted. Data were analyzed and graphed using GraphPad Prism 5.0 statistical analysis software (version 5.0, GraphPad Software, La Jolla, CA, USA). Results are expressed as means ± standard error mean. All data were tested for normality and analyzed by one-way ANOVA and post hoc Tukey test as necessary. A *p* value < 0.05 was used to determine significance.

## Abbreviations

*Cd11b*	Cluster of differentiation 11b
*Cd68*	Cluster of differentiation 68
EWAT	Epididymal white adipose tissue
HFD	High fat diet
IL-6	Interleukin 6
IWAT	Inguinal white adipose tissue
*Mcp-1*	Monocyte chemoattractant protein 1
ROE	Red onion extract
